# FimL Regulates cAMP Synthesis in *Pseudomonas aeruginosa*

**DOI:** 10.1371/journal.pone.0015867

**Published:** 2011-01-11

**Authors:** Yuki F. Inclan, Medora J. Huseby, Joanne N. Engel

**Affiliations:** 1 Department of Medicine, University of California San Francisco, San Francisco, California, United States of America; 2 Department of Microbiology and Immunology, University of California San Francisco, San Francisco, California, United States of America; 3 Microbial Pathogenesis and Host Defense Program, University of California San Francisco, San Francisco, California, United States of America; Charité-University Medicine Berlin, Germany

## Abstract

*Pseudomonas aeruginosa*, a ubiquitous bacteria found in diverse ecological niches, is an important cause of acute infections in immunocompromised individuals and chronic infections in patients with Cystic Fibrosis. One signaling molecule required for the coordinate regulation of virulence factors associated with acute infections is 3′, 5′-cyclic adenosine monophosphate, (cAMP), which binds to and activates a catabolite repressor homolog, Vfr. Vfr controls the transcription of many virulence factors, including those associated with Type IV pili (TFP), the Type III secretion system (T3SS), the Type II secretion system, flagellar-mediated motility, and quorum sensing systems. We previously identified FimL, a protein with histidine phosphotransfer-like domains, as a regulator of Vfr-dependent processes, including TFP-dependent motility and T3SS function. In this study, we carried out genetic and physiologic studies to further define the mechanism of action of FimL. Through a genetic screen designed to identify suppressors of FimL, we found a putative cAMP-specific phosphodiesterase (CpdA), suggesting that FimL regulates cAMP levels. Inactivation of CpdA increases cAMP levels and restores TFP-dependent motility and T3SS function to *fimL* mutants, consistent with *in vivo* phosphodiesterase activity. By constructing combinations of double and triple mutants in the two adenylate cyclase genes (*cyaA* and *cyaB*), *fimL*, and *cpdA*, we show that Δ*fimL* mutants resemble Δ*cyaB* mutants in TM defects, decreased T3SS transcription, and decreased cAMP levels. Similar to some of the virulence factors that they regulate, we demonstrate that CyaB and FimL are polarly localized. These results reveal new complexities in the regulation of diverse virulence pathways associated with acute *P. aeruginosa* infections.

## Introduction

*Pseudomonas aeruginosa* is an important human pathogen found throughout the environment in diverse ecological niches and is associated with severe opportunistic infections [1]. Patients with some degree of immunocompromise and epithelial barrier injury, such as those with severe burns or surgical incisions, on mechanical ventilation, or receiving cytotoxic chemotherapy, are particularly vulnerable to acute infection. This Gram-negative bacterium is also the leading cause of chronic pulmonary infections and death in Cystic Fibrosis patients. Even with appropriate medical treatment, mortality remains high and antibiotic resistance is increasingly common. Thus, it is of great importance to identify new potential therapeutic targets.

*P. aeruginosa* produces several virulence factors associated with acute infection, including components of the Type IV pilus (TFP), Type III secretion system (T3SS), Type II secretion system (T2SS), and quorum sensing systems (reviewed in [2]). TFP are polarly localized appendages composed of pilin polymers that undergo reversible assembly and disassembly and allow the bacteria to move over a solid surface in a process termed twitching motility (TM) (reviewed in [3]). In addition, TFP function as phage receptors, are initiators of biofilm formation, and are the primary adhesins associated with *P. aeruginosa* binding to both biotic and abiotic surfaces, including mammalian cells. The T2SS functions to secrete enzymes such as proteases, hemolysins and toxins into the extracellular milieu to cause host cell cytotoxicity. The T3SS, which resembles a molecular syringe, directly injects effector proteins, including ExoU, ExoS, ExoT, and ExoY, from the bacteria to the host cell cytosol. Components of related T3SS in *Shigella flexneri* and *Yersinia pestis* have been shown to localize to the cell pole [4], [5]. These effector proteins have profound outcomes on host cell signaling, leading to host cell death and alterations in the host immune system. The bacterial molecules involved in the activation and coordination of virulence factor production include numerous signal transduction cascades [6].

One signaling molecule required for the coordinate regulation of virulence factors associated with acute infections is 3′, 5′-cyclic adenosine monophosphate, (cAMP). cAMP functions as a co-factor for the cAMP binding protein, variously known as CAP or CRP. Upon binding cAMP, CRP undergoes a conformational change and binds to specific DNA sequences, thus modulating gene transcription. cAMP signaling has also been shown to regulate a variety of processes in many bacteria, from iron uptake in *E. coli* [7] to competence in *Haemophilus influenzae* [8]. cAMP signaling in the regulation of the *lac* operon during diauxic growth [9] and in catabolite repression [10] has been extensively studied in *Escherichia coli* and other bacteria. More recently, cAMP signaling has been implicated in the regulation of virulence factors for many pathogenic *firmicutes* and *proteobacteria*, although the mechanisms vary widely [10].

In *P. aeruginosa*, cAMP is an allosteric regulator of the CRP homolog, Vfr (virulence factor regulator) [11]. In contrast to *E. coli*, cAMP signaling in *P. aeruginosa* is not involved in catabolite repression. Instead, Vfr controls the transcription of over 200 genes, including those associated with TFP, T3SS, T2SS, flagellar-mediated motility, and quorum sensing systems [12], [13]. The mechanism by which Vfr regulates all of these virulence factors is not entirely known. A consensus DNA binding sequence has been identified but does not fully explain every gene regulated by Vfr [14]. To add further complexity, recent data suggests that Vfr may control expression of some genes in a cAMP-independent manner [15].

cAMP levels are regulated by synthesis and degradation [16], [17]. In *P. aeruginosa*, two adenylate cyclases have been described that synthesize cAMP. CyaB, a membrane bound adenylate cyclase, plays the major role in cAMP synthesis, while CyaA, a cytoplasmic adenylate cyclase, plays a minor role in synthesis [12], [18]. Accordingly, CyaB is an important regulator of Vfr-dependent functions; Δ*cyaB* and Δ*vfr* mutants exhibit attenuated virulence in a mouse model of acute pneumonia infection [19].

We previously carried out a transposon-mediated genetic screen to identify *P. aeruginosa* genes important for host cell cytotoxicity. This screen identified TFP, ExoU, and T3SS components as critical mediators of host cell death [20], [21]. Subsequent studies have also shown that TFP and the T3SS are important virulence factors in mouse models of acute *P. aeruginosa* infections [20], [22], [23] and that the presence of a functional T3SS correlates with unfavorable clinical outcomes in acute human infections [24].

Our genetic screen also identified a protein, FimL, with homology to the N-terminus of a *P. aeruginosa* CheA homolog, ChpA [25]. In addition to the hybrid kinase ChpA, the Chp operon encodes two CheY homologs as well as other components of the chemosensory system and regulates pilin extension and retraction [26]. We demonstrated that FimL was important in regulating TFP function, production and secretion of T3SS associated virulence factors, biofilm formation, and concluded that FimL likely functions upstream of Vfr. Spontaneous extragenic suppressors of FimL, which arose at higher than expected frequencies, regained TM and T3SS-mediated cytotoxicity towards mammalian cells. One suppressor mutant was analyzed in greater detail and found to produce nearly ten-fold higher levels of cAMP compared to the parent strain [25] although the site of the extragenic mutation(s) remains unknown. Together, these results suggest that FimL and Vfr are components of intersecting pathways that serve to regulate diverse virulence factors involved in acute infections.

In this study, we performed genetic and physiologic experiments to further define the mechanism of action of FimL. In a transposon mutant screen designed to identify extragenic suppressors of FimL, we found a cAMP-specific phosphodiesterase, suggesting that FimL regulates cAMP levels. We measured cAMP levels in the Δ*fimL* mutant and found decreased levels of cAMP, similar to the diminished levels observed in Δ*cyaB*. By constructing informative double and triple mutants in *cyaA*, *cyaB*, *cpdA* and *fimL*, we show that Δ*fimL* mutants resemble Δ*cyaB* mutants with respect to altered TM, decreased T3SS transcription, and decreased cAMP levels. Epistasis experiments suggest that FimL function is CyaB dependent. Similar to some of the virulence factors regulated by cAMP, including TFP [27], flagella [28], and potentially the T3SS, FimL and CyaB are polarly localized. While this manuscript was in preparation, it was reported that FimL and other components of the Chp operon also modulate cAMP levels [29]. Together, these results suggest that the Chp system not only regulates pilin function but also coordinately regulates cAMP-dependent pathways, including the T3SS. We hypothesize that FimL plays a pivotal role in this coordinate regulation by interfacing with the Chp chemosensory system, TFP, and the cAMP/Vfr pathway. Our studies reveal new complexities in the regulation of diverse virulence pathways associated with acute *P. aeruginosa* infections.

## Materials and Methods

### Bacterial Cultures and Assays

The bacterial strains and plasmids used in this study are described in Table S1. Bacteria were routinely streaked onto on Luria-Bertani (LB) 1.5% agar and grown in liquid LB overnight shaking at 250 rpm at 37°C. *E. coli* strains S17.1 and SM10 were used as donor strains with *P. aeruginosa* recipient strains in bacterial conjugation. After mating with *E. coli*, *P. aeruginosa* strains were selected by growth on 1.5% Difco Pseudomonas isolation agar (Becton Dickinson). Antibiotic concentrations used for *E. coli*, tetracycline, 5 µg/mL; ampicillin, 100 µg/mL; gentamicin, 10 µg/mL; kanamycin 50 µg/mL and for *P. aeruginosa*, tetracycline, 100 µg/mL, carbenicillin 250 µg/mL, gentamicin, 100 µg/mL. For β-galactosidase experiments and cAMP assays, bacterial frozen stocks were freshly streaked onto LB agar, and following overnight growth were inoculated into 5 mL of MinS media [30] with or without addition of CaCl_2_ to a final concentration of 2 mM. Following growth overnight, samples were processed.

*β-galactosidase assays* were performed as previously described [25].

*cAMP assay*s were performed using the Cayman cAMP EIA assay kit. Briefly, a volume of culture corresponding to 10 OD600 units was pelleted, and resuspended in 500 µL 0.1 M HCl. Following 30 seconds of sonication, the debris was removed with a low-speed spin after 10 min at 1000 X G for PA103 or 14,000 X G for PAO1 strains. 400 µL of supernatant was transferred to a fresh tube containing an equal volume of EIA buffer (supplied with kit), and the samples were further processed according to the manufacturer's manual. For acetylated reactions, samples were processed as above, except that the 500 µL of supernatant (following sonication and pelleting) was acetylated according to manufacturer's protocol prior to dilution in EIA buffer and further processing.

### Transposon Mutagenesis

The donor strain SM10-λ pir containing pBT20 was mated with the recipient strain PAO1Δ*fimL*::CTXP*_exoT_-lacZ*. Transformants were plated on 1.5% LB agar plates containing bromo-chloro-indolyl-galactopyranoside (40 µg/mL) and gentamicin and screened for restoration of TM and blue colony color. Insertion sites were determined using semirandom PCR [31].

*Twitching Motility assays* were performed using the subsurface stab assay as previously described [26].

### Plasmid construction

All plasmids were purified using Qiagen kits and standard molecular biology recombinant techniques were used. Enzymes were purchased from New England Biolabs and used as recommended by the manufacturer. All primers used in construction were designed using the PAO1 genome (www.pseudomonas.com) and synthesized by Qiagen or Elim Biopharmaceuticals, Inc. Primer sequences are available upon request.

### Construction and complementation of in-frame deletion mutants

All matings were performed as described previously [26]. In general, a PCR fragment 1 kb upstream of the target deletion gene and 1 kb downstream was synthesized and overlap PCR performed to create the deletion PCR fragment. This fragment with created with unique restriction enzyme sites on both ends and cloned into pJB100T or pEX100T. This plasmid was then introduced into S17.1 *E. coli* strain and mated to *P. aeruginosa*. The vector backbone was then counter-selected using 5% sucrose-containing 1.5% LB agar plates and the resulting unmarked deletion strain was checked with PCR and/or Southern blot.

### Construction of CTX-exoT-lacZ strains

The promoter region (600 bp upstream of the *exoT* gene) was directionally cloned into the mini-CTX-*lacZ* vector as a HindIII-EcoR1 fragment, following amplification from genomic DNA with the primers 5′AAGCTTCCACGCCTGACATCGCTCAC 3′ and 5′GAATTCGCCACGAAAGACGGGTTCTG 3′. The resulting CTX-P*_exoT_*-*lacZ* plasmid was mated into wild type PAO1 to generate PAO1::CTX*P_exoT_-lacZ*. The Δ*fimL* derivative was generated by mating the Δ*fimL* allelic exchange plasmid pJEN36 into PAO1::CTX*P_exoT_-lacZ*. The Δ*vfr* derivative was generated by mating pJEN51 into PAO1::CTX*P_exoT_-lacZ*. PA103 versions of these strains were also generated with these plasmids.

### Construction of chromosomal and plasmid-borne FimL3X-FLAG strains

A C-terminal triple FLAG-tagged version of *fimL*, containing a diagnostic Cla1 restriction site was constructed as follows. Using primers 5′CGGGATCCCCGGCCCGGCCAGCCATAGCAGCAGGGGG-3′ and 5′CCATCGATTTTATCGTCATCGTCTTTGTAGTCGGCGGCTTTATCGTCATCGTCTTTTAGTCGGCGGCCACCGGCAGTCCGA -3′, a 3 kb fragment containing a BamH1 site followed by 1 kb of sequence upstream of *fimL*, the *fimL* coding region immediately upstream of the stop codon, and 2 FLAG tags followed by Cla1 site was amplified. Separately, primers 5′CCATCGATGACTACAAAGATGACGATGACAAATGATGGCCGGCGAGTTCCGCTGGC-3′ and 5′- GCTCAGACGGAGCGTTCTGGACCGTGACCTC-3′ were used to amplify a fragment containing a Cla1 site, followed by a single FLAG tag, the stop codon, and 1 kb of sequence downstream of *fimL*, terminating with an Xba1 site. The amplicons were digested respectively with BamH1 and Cla1, and Cla1 and Xba1, ligated together into pOK12 that had been digested with BamH1 and Xba1. The resulting triple FLAG-tagged *fimL* fragment was excised with Spe1 and cloned into the allelic exchange vector pJB100T. The resulting plasmid pJTW019 was mated into PAO1Δ*fimL*::CTXP*_exoT_-lacZ*. The complemented *fimL* mutant was confirmed to carry the triple flag-tagged fimL by PCR/Cla1 digest, and functional complementation was confirmed by Western blot using an anti-Flag monoclonal antibody (Sigma). The insert from pJW019 was subcloned as a Spe1 fragment into the Xba1 site of pUCP19Δ*lac*. A BamH1 digest was performed to identify which clones were in the correct orientation to be expressed from the pTac promoter. The resulting plasmid pUCP19-*fimL3X-FLAG* was transformed into *P. aeruginosa* strains (see Table S1); expression from the plasmid was confirmed by Western blot.

### Construction of FimL-GFP strains

*fimL* was PCR amplified using 5′ TGGGCTAGCGAATTCATGGTCACAGGAGCC and 5′ GGACTGCCGGTGGCCGCCGGCGGCGGCAAGCTTGTGAGCAAG with a 3XGly linker, minus the STOP codon and cloned into pMBAD-GFP to generate pYFI007. *fimL-GFP* was subcloned from pYFI007 into PJB100T by overlap PCR with a PCR fragment downstream of *fimL* on the PAO1 chromosome and was amplified using primers 5′ CGGCATGGACGAGCTGTACAAGTAATGGCCGGCGAGTTCCGCTGGC and 5′CAGGGTAATACTAGTAGCGGCGCGCCAGGTAC to generate the *fimL-GFP* gene replacement construct pYFI043. Similarly, *cyaB* was amplified using 5′ GCTAGCGAATTCATGAAGCCTACCCTCCCCGACC 3′ and5′ CCCCGGGTACCGCCGCCGCCGAGGATGACCTTGTCGCGCAGG3′ to generate pYFI184.

*SDS-PAGE and immunoblotting* assays were performed as in [32].

## Results

### A screen for extragenic suppressors of fimL identifies the phosphodiesterase CpdA

To further understand how FimL controls virulence pathways, we devised a genetic screen to identify new regulators in the FimL pathway. Our goal was to find single gene disruptions that resulted in restoration of TFP function and T3SS function in a Δ*fimL* mutant. Therefore, we screened a library of transposon insertions in a Δ*fimL* strain for mutants that simultaneously regained TM and T3SS function. Specifically, we utilized a mariner transposon [33] to mutagenize PAO1Δ*fimL* containing an *E. coli lacZ* gene reporter fused to the promoter of the T3SS effector *exoT* (denoted *P_exoT_-lacZ*). Approximately 100,000 transposon mutants (approximately 20X genome coverage) were visually screened on LB plates containing bromo-chloro-indolyl-galactopyranoside (X-gal) and isopropyl β-D-1-thiogalactopyranoside (IPTG) for simultaneous recovery of *exoT* transcription (as evidenced by blue colonies) and TFP function (as evidenced by TM, a phenotype readily visible as a colony with an expanding edge). From this screen, we recovered a transposon insertion in PA4969, which regained *P_exoT_-lacZ* expression and TM in the Δ*fimL* background. We constructed an in-frame deletion of PA4969 in PAO1Δ*fimL* (PAO1Δ*fimL*Δ*cpdA*) and confirmed that inactivation of PA4969 was responsible for suppressing the Δ*fimL* phenotype. PAO1Δ*fimLΔ*Δ*cpdA* showed high *P_exoT_-lacZ* expression (Fig. S1A) and partially restored TM (Fig. S1B). During the preparation of this manuscript, PA4969 was characterized as a cAMP- specific phosphodiesterase, CpdA [34].

### FimL regulates cAMP levels

**Figure 1:**
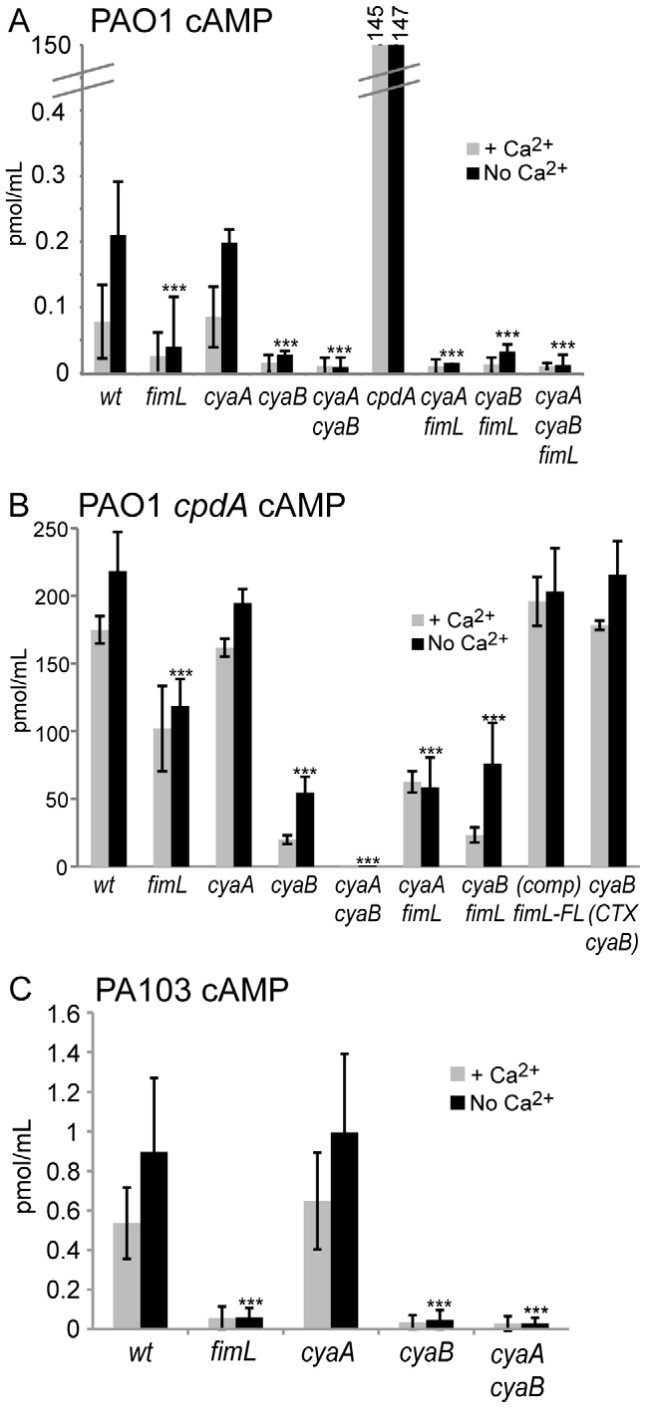
cAMP levels are decreased in *fimL* mutants. **Intracellular** cAMP levels were measured in the presence (2 mM) or absence of calcium in (A) PAO1, (B) PAO1Δ*cpdA*, or (C) PA103. Gene names denote in-frame deletions. PAO1Δ*cpdA*(comp) *fimL-FL* denotes complementation of PAO1Δ*cpdA*Δ*fimL* with *fimL-3X-FLAG* by gene replacement in the *fimL* locus. PAO1Δ*cpdA*Δ*cyaB* (CTX-*cyaB*) denotes complementation of PAO1Δ*cpdA*Δ*cyaB* with *cyaB* at the CTX phage attachment site. Note the scale differences. Shown are mean results of triplicate samples from 3 experiments. Error bars indicate SD (for *cpdA* in (A) +/−4.7 in the presence of Ca^2+^ and +/−3.6 in the absence of Ca^2+^). (***) P<0.001 compared to the wild type strain grown in the absence of calcium.

Since disrupting *cpdA* restored TM and T3SS function in the Δ*fimL* mutant, we predicted that Δ*fimL* mutants would have decreased levels of cAMP. To test this hypothesis, we measured bacterial cAMP. The experiments were performed in the presence and absence of calcium, since minimal media lacking calcium induces expression of T3SS gene expression [35]. We previously reported that cAMP levels were barely detectable in the wild type PAO1 strain [25], however with protocol changes we were able to reproducibly measure cAMP in PAO1. In the absence of calcium, we observed an ∼80% decrease in cAMP levels in the Δ*fimL* mutant compared to PAO1 (Fig. 1A; P<0.001). As controls, we assayed PAO1Δ*cyaA*, PAO1Δ*cyaB*, and the double mutant PAO1Δ*cyaA*Δ*cyaB*. No significant difference was observed in cAMP levels in the Δ*cyaA* strain when compared to wild type. However the Δ*cyaB* and Δ*cyaA*Δ*cyaB* mutants showed an ∼85% and ∼95% decrease, respectively, in cAMP levels when compared to wild-type (P<0.001). The low cAMP levels observed in PAO1Δ*cyaB*, PAO1Δ*fimL* and PAO1Δ*cyaA*Δ*cyaB* were not statistically different from each other. Together, these results suggest that FimL modulates cAMP levels.

Deletion of the phosphodiesterase gene (PAO1Δ*cpdA*) resulted in an approximately 100-fold increase in cAMP levels compared to wild type (Fig. 1A). Thus we reasoned that we could detect changes in cAMP levels very robustly in a sensitized mutant background in which *cpdA* was inactivated. We therefore constructed in-frame deletions of *fimL*, *cyaA*, and *cyaB* in the phosphodiesterase mutant background PAO1Δ*cdpA* and measured cAMP levels. As shown in 1B, deletion of *fimL* (PAO1Δ*cpdA*Δ*fimL*) or *cyaB* (PAO1Δ*cdpA*Δ*cyaB*) resulted in ∼50% (P<0.001) and ∼75% (P<0.001) decrease in cAMP levels, respectively, compared to PAO1Δ*cdpA,* while no statistically significant decrease was observed in the *cyaA* mutant (PAO1Δ*cpdA*Δ*cyaA*). cAMP was undetectable in the PAO1Δ*cpdA*Δ*cyaA*Δ*cyaB* mutant, confirming that CyaA and CyaB are the only source of cAMP in this strain. Altogether, the trends in cAMP levels in PAO1 or the PAO1Δ*cpdA* background are similar, lending further support to our conclusion that FimL regulates cAMP levels.

To ascertain if these results were strain-specific, we constructed in-frame deletion mutants in PA103. We reproducibly detected ∼10-fold decrease in cAMP levels in PA103Δ*fimL* and PA103Δ*cyaB* compared to PA103 (Fig. 1C; P<0.001). The levels of cAMP were restored to near wild type levels in the PA103Δ*fimL* strain complemented with FimL-3X-FLAG at the endogenous locus (Fig. S2). As expected, in PA103Δ*cyaA*, cAMP levels were indistinguishable from wild type PA103, and cAMP was undetectable in the PA103Δ*cyaA*Δ*cyaB* mutant (Fig. 1C). Thus, deletion of *fimL* correlates with a decrease in cAMP levels in both PAO1 and PA103, strongly supporting a role for FimL in cAMP production in *P. aeruginosa*.

### FimL promotes CyaB-dependent cAMP synthesis

FimL regulation of cAMP could occur by affecting cAMP synthesis or by stimulating degradation of cAMP via the phosphodiesterase CpdA. If FimL only functions through CpdA activity, then cAMP levels should not change between the Δ*cpdA* and the Δ*cpdA*Δ*fimL* mutants. As shown in Fig. 1B, cAMP levels are reduced in PAO1Δ*cpdA*Δ*fimL* compared to PAO1Δ*cpdA*, suggesting that FimL regulates cAMP independent of CpdA.

We therefore considered the alternative model that FimL regulates cAMP biosynthesis and compared cAMP levels in the presence or absence of FimL in PAO1Δ*cyaA*, PAO1Δ*cyaB*, and PAO1Δ*cyaA*Δ*cyaB* (Fig. 1A). cAMP levels decreased ∼90% in PAO1Δ*cyaA*Δ*fimL* compared to PAO1Δ*cyaA* (P<0.001). In contrast, there was no statistically significant difference in cAMP levels between PAO1Δ*cyaB* and PAO1Δ*cyaB*Δ*fimL* or between PAO1Δ*cyaA*Δ*cyaB* and PAO1Δ*cyaA*Δ*cyaB*Δ*fimL*. In the Δ*cyaA*Δ*fimL* mutant, CyaB is the only source of cAMP; thus, the decrease in cAMP levels in this mutant compared to the Δ*cyaA* mutant suggests that FimL affects cAMP synthesis via CyaB. The finding that cAMP levels are indistinguishable between Δ*cyaB*Δ*fimL* and Δ*cyaB* also suggests that FimL does not act through CyaA. Similar results were observed with deletion mutants in the PAO1Δ*cpdA* background (Fig. 1B).

### FimL does not affect cyaB transcription or protein levels

**Figure 2:**
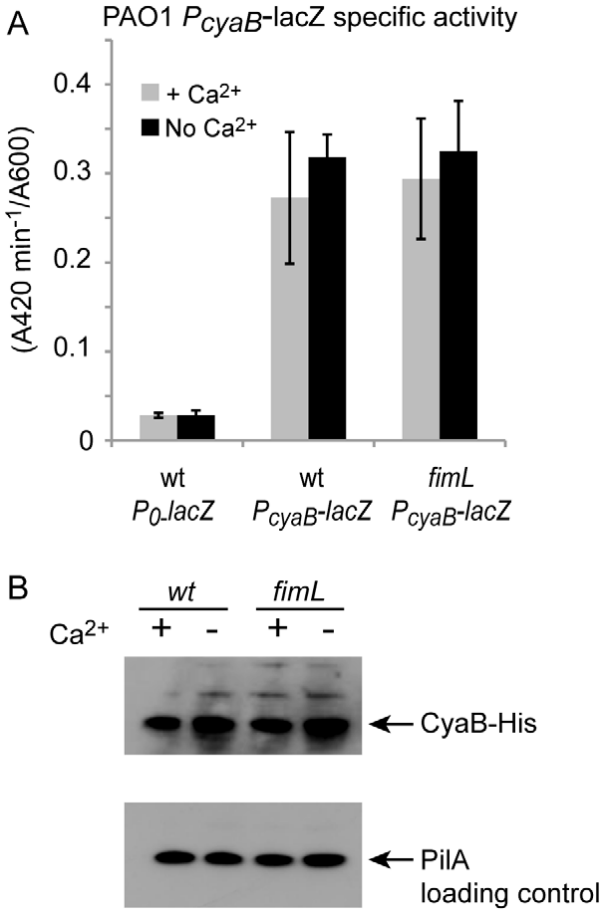
Loss of *fimL* does not affect *cyaB* transcription or protein levels. (A) β-galactosidase activity was measured in the presence or absence of calcium in PAO1 or PAO1Δ*fimL*. All strains carry a *lacZ* transcriptional reporter integrated at the CTX site without a promoter (P*_0_-lacZ*) or with the *cyaB* promoter (Pc*yaB-lacZ*). Error bars indicate SD of the average rate from 14 data points taken from two experiments. (B) Immunoblot of PAO1 or PAO1Δ*fimL* in which the wild type *cyaB* gene has been replaced with *cyaB-His*. Lysates were prepared from bacteria grown in the presence or absence of Ca^2+^ and probed with anti-His antibody (upper panel) or anti-PilA antibody (lower panel) as a loading control.

Our results so far suggest that FimL modulates cAMP levels through CyaB. This regulation could occur by modulating *cyaB* transcription, CyaB protein levels, or CyaB activity. To determine whether *cyaB* expression was altered at the transcriptional level, we compared the β-galactosidase activity of the *lacZ* gene fused to the promoter of *cyaB* and integrated into the chromosome at the CTX site in PAO1 and PAO1Δ*fimL*. As shown in Fig. 2, there was no difference in transcription from the *cyaB* promoter in the wild type or the Δ*fimL* background. Minimal transcription of the *lacZ* reporter was observed in the absence of the *cyaB* promoter. In contrast to strain PAK in which *cyaB* transcription was reported to be calcium dependent in microarray analysis [12], we did not observe calcium-dependent *cyaB* transcription in PAO1.

We next assayed CyaB protein production in a strain in which the native *cyaB* gene was replaced with a *cyaB-His* fusion. As shown in Fig. 2B, protein levels of CyaB-His were unaltered in PAO1Δ*fimL*. We therefore conclude that FimL modulates CyaB function at a post-translational step.

### Expression of T3SS genes correlates with intracellular cAMP levels

We previously published that *fimL* mutants exhibited decreased ExoT production and secretion [25]. *vfr* mutants were also shown to be defective in T3SS function [12]. Together with published data and given our cAMP results, we predicted that *fimL* mutants would show decreased transcription of T3SS genes. We used our panels of isogenic mutants to directly assess the role of FimL and cAMP on the transcription of a representative T3SS gene, the secreted toxin ExoT. This readout is robust and can readily be detected in either PA103 or PAO1.

**Figure 3:**
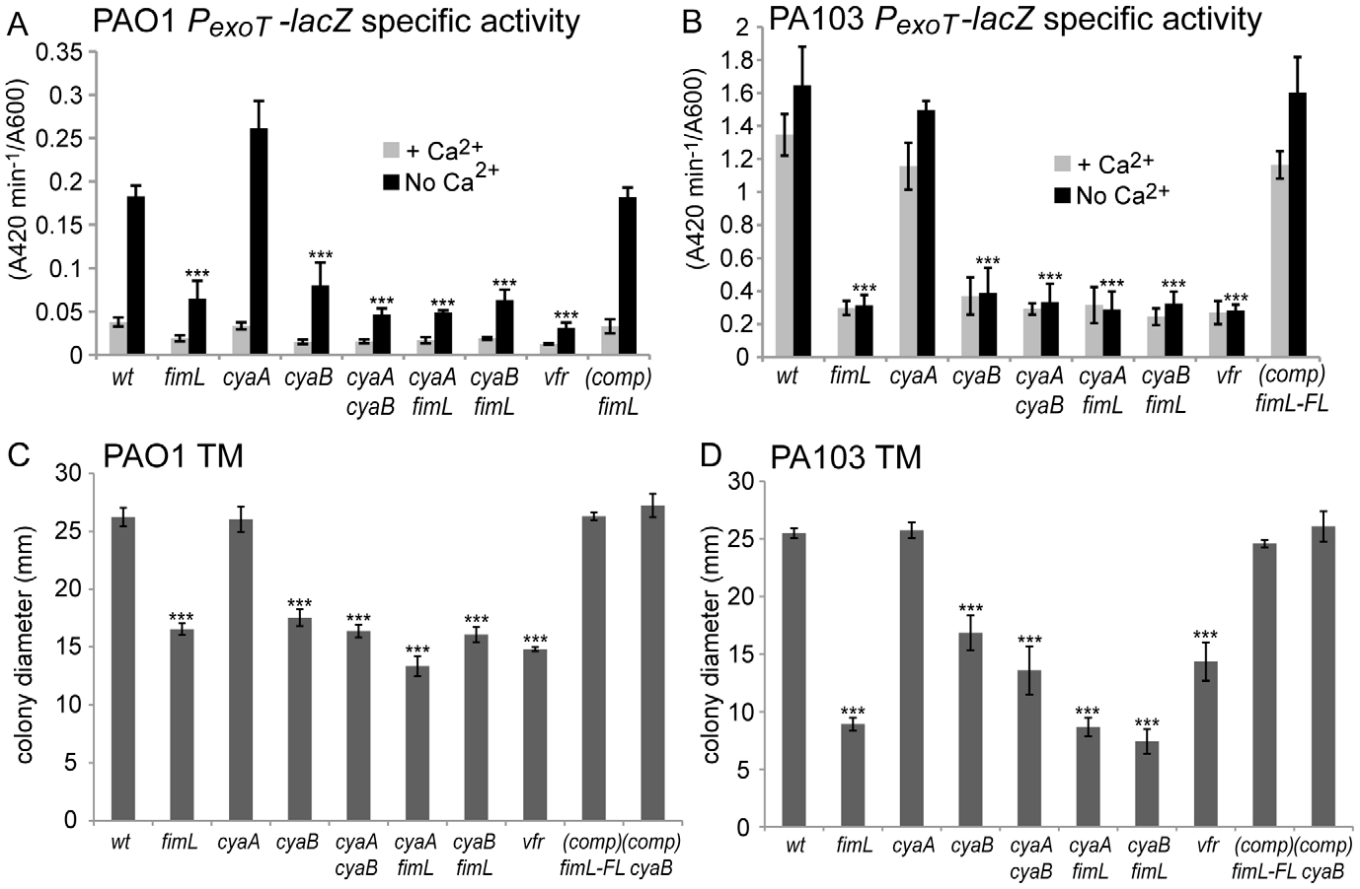
FimL, CyaB, and Vfr are required for *exoT* transcription and TM. All strains harbor the P*exoT-lacZ* transcriptional reporter fusion integrated at the CTX site as a readout for transcription of the T3SS. β-galactosidase activity was measured in the presence or absence of calcium. Gene names denote in-frame deletions in PAO1 (A and C) or PA103 (B and D). (A and B) Shown are mean of 12 data points from triplicate samples from 2 or 3 experiments. (C and D). Shown is the mean diameter from a minimum of 5 colonies from 2 or 3 experiments. Error bars denote SD. (***) indicates P<0.001 compared to the wild type strain grown in the absence of Ca^2+^.

A transcriptional fusion comprising the *exoT* promoter fused to *lacZ* (*P_exoT_-lacZ*) was integrated at the CTX phage attachment site in Δ*fimL*, Δ*cyaA*, and Δ*cyaB* mutants in PAO1 (Fig. 3A) and PA103 (Fig. 3B) and assayed for β-galactosidase activity in the presence or absence of calcium. We reproducibly found a correlation between cAMP levels and *P_exoT_-lacZ* transcription. Compared to wild type PAO1 or PA103, *P_exoT_-lacZ* transcription was unaffected in the PAO1Δ*cyaA* mutant or PA103Δ*cyaA* mutant, respectively. In contrast, the corresponding Δ*fimL* and the Δ*cyaB* mutants showed greatly reduced *P_exoT_-lacZ* expression, similar to the levels seen in the Δ*vfr* and Δ*cyaA*Δ*cyaB* mutants. *P_exoT_-lacZ* transcription in PAO1Δ*fimL* and PA103Δ*fimL* was restored to wild type levels upon complementation with *fimL* (Figs. 3A and 3B).

### TM correlates with cAMP levels

Assembly and function of TFP requires over 40 gene products whose transcription is regulated in part by Vfr [3], therefore we predicted that cAMP would also regulate TM. We measured TM of Δ*cyaA*, Δ*cyaB*, Δ*fimL* and Δ*vfr* mutants in PAO1 and PA103 by the subsurface stab assay. All mutants that exhibited decreased cAMP levels (Fig. 1) and decreased *P_exoT_-lacZ* expression (Figs. 3A and 3B) also showed reduced TM, including Δ*fimL*, Δ*cyaB*, Δ*cyaA*Δ*fimL*, Δ*cyaB*Δ*fimL*, and Δ*cyaA*Δ*cyaB* mutants in both the PAO1 (Fig. 3C) and PA103 (Fig. 3D) backgrounds. TM was restored to the wild type diameter upon complementation of the corresponding Δ*fimL* mutant.

### Ectopic expression of fimL reduces cAMP levels

**Figure 4:**
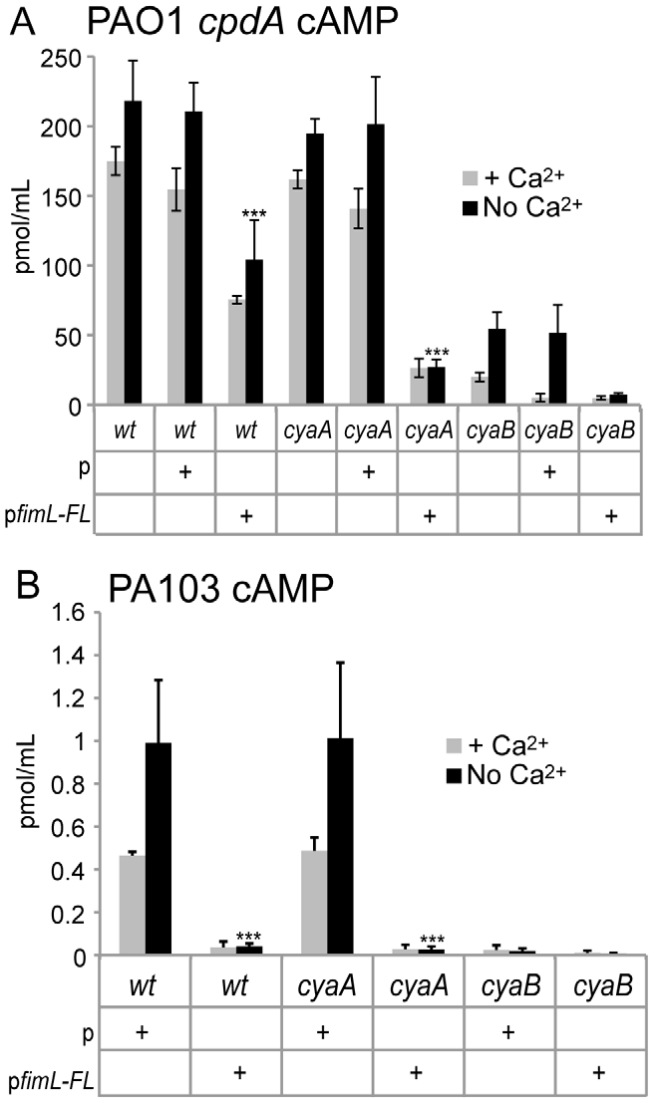
Ectopic expression of *fimL* decreases cAMP. cAMP levels were measured in (A) PAO1Δ*cpdA* or (B) PA103 with p (empty vector) or p*fimL-FL* (vector with *fimL-3X-FLAG*). Shown are mean values of 3 experimental repetitions performed in triplicate. Error bars indicate SD. (***) P<0.001 compared to the respective strains containing vector only and grown in the absence of Ca^2+^.

We tested the effect of ectopic expression of *fimL* on cAMP levels and cAMP-dependent phenotypes. FimL-FLAG was cloned into a plasmid under the control of a constitutive promoter (pUCP19Δ*lac*), denoted “p*fimL*-*FL*” and introduced into PAO1, PAO1Δ*cpdA*, and PA103. We confirmed that the addition of the epitope tag did not affect FimL function by demonstrating that *fimL-FL* restored cAMP production in PAO1Δ*cpdA* (Fig. 1B) and in PA103Δ*fimL* (Fig. S2). Expression of the empty vector had no effect on cAMP levels; however, expression of p*fimL-FL* resulted in decreased levels of cAMP in PAO1Δ*cpdA* and PAO1Δ*cpdA*Δ*cyaA* compared to the corresponding isogenic strain containing the control vector (Fig. 4A). Similar results were observed when p*fimL*-*FL* was introduced into PA103 (Fig. 4B) and PAO1 (data not shown) suggesting that the decreased cAMP levels upon ectopic expression of *fimL* are not strain-specific. Expression of p*fimL-FL* did not affect *P_cyaB_-lacZ* expression (Fig. S3), indicating that the inhibitory effect of FimL is not through *cyaB* transcription. Expression of p*fimL-FL* in PAO1Δ*cyaB* or PA103Δ*cyaB* did not significantly change the already low levels of cAMP in these strains.

**Figure 5:**
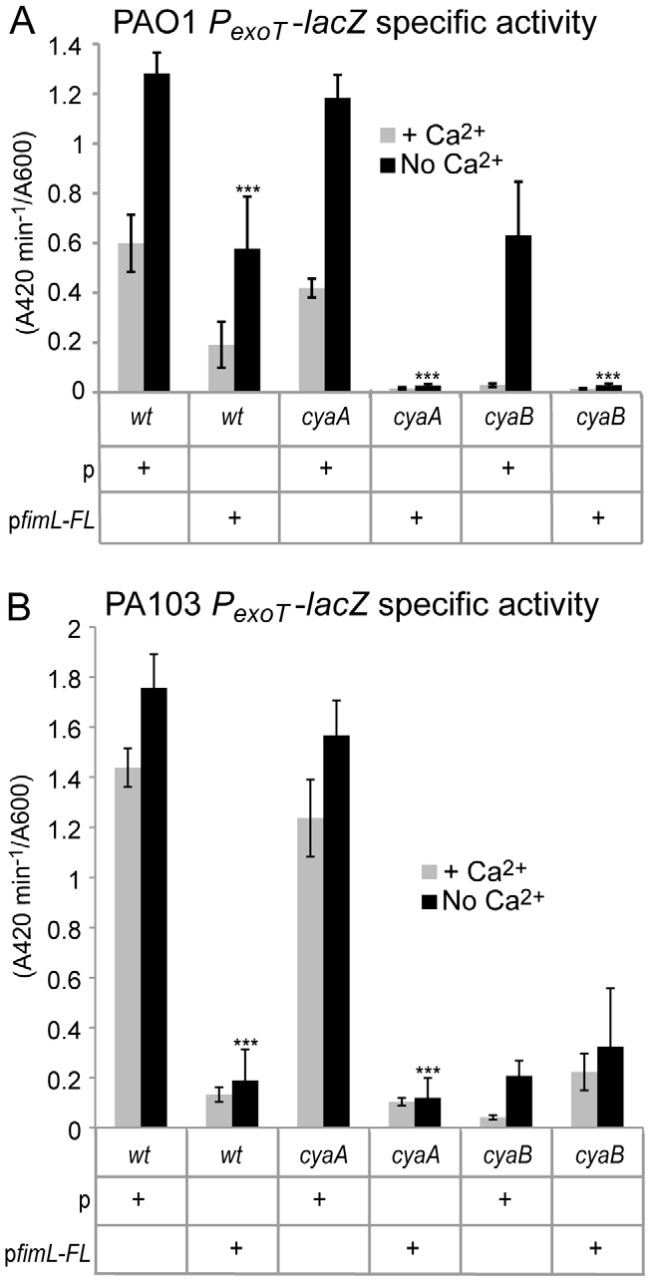
Ectopic expression of *fimL* inhibits *exoT* transcription. All strains harbor the P*exoT-lacZ* transcriptional reporter fusion integrated at the CTX site as a readout for transcription of the T3SS and either include p (vector only) or p*fimL-FL* (vector with *fimL-3X-FLAG*). β-galactosidase activity was measured in the presence or absence of Ca^2+^ in the indicated mutants in (A) PAO1 or (B) PA103. Shown is the mean of 12 data points from 3 or 4 experiments. Error bars indicate SD. (***) P<0.001 compared to the respective strain containing vector only grown in the absence of Ca^2+^.

Given these findings, we predicted that ectopic expression of *fimL* would also affect TM and transcription of T3SS genes. As shown in Fig. S4, ectopic expression of p*fimL-FL* inhibited TM in PAO1. Likewise, ectopic expression of p*fimL-FL* decreased *P_exoT_-lacZ* expression in PAO1 (Fig. 5A) and PA103 (Fig. 5B) and in the corresponding Δ*cyaA* mutants. As expected from previous experiments, ectopic expression of p*fimL-FL* in PA103Δ*cyaB* did not further decrease the already low levels of *P_exoT_-lacZ* transcription, supporting the hypothesis that the regulation of cAMP by FimL is CyaB-dependent.

Together, these results suggest that precisely tuned levels of FimL are critical to modulate cAMP levels. It is possible that in order to promote cAMP synthesis efficiently, the stoichiometry of FimL:CyaB is critical. However, FimL is not absolutely required for CyaB function, as some cAMP is synthesized in the absence of FimL (Fig. 1). Consistent with these observations, we found that expression of p*cyaB* can complement the PAO1Δ*fimL* mutant for T3SS function (Fig. S5). We propose that FimL is required for optimal CyaB adenylate cyclase activity.

### FimL and CyaB are polarly localized

**Figure 6:**
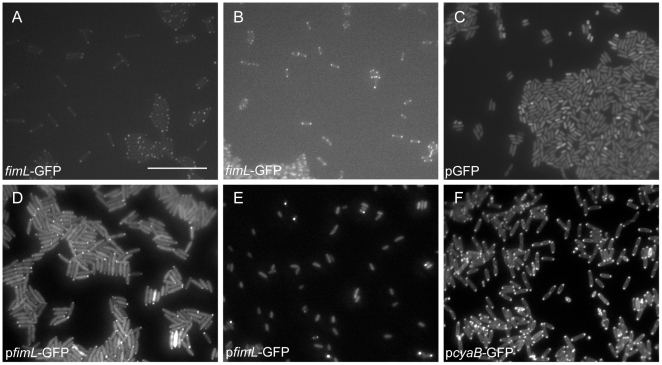
FimL and CyaB are polarly localized. Images of (A) log-phase or (B) stationary phase grown PAO1 *fimL-GFP* in which *fimL-gfp* replaced the native *fimL* gene. Scale bar is approximately 10 µm. (C) PAO1 GFP with p*GFP* (stationary phase). (D) PAO1 Δ*fimL* + p*fimL-GFP* without arabinose induction (log phase). (E) PAO1 + p*fimL-GFP* with arabinose induction (stationary phase). (F) PAO1 Δ*cyaB* + p*cyaB-GFP* with arabinose induction (log phase).

Many of the virulence factors that are regulated by FimL, such as TFP and potentially the T3SS, are polarly localized in *Enterobacteriaceae*. We therefore investigated the localization of FimL and CyaB. For our initial studies, we constructed a plasmid-borne *fimL-GFP* fusion under control of an arabinose inducible promoter (p*fimL*-*GFP*) and introduced the plasmid into PAO1 or PAO1Δ*fimL*. In the absence of arabinose induction, low-level expression of *fimL-GFP* was sufficient to restore TM in PAO1Δ*fimL* (Fig. S6A), confirming that fusion to GFP did not interfere with FimL function. When *fimL-GFP* was induced with arabinose, TM and cAMP levels were inhibited compared to the control strain carrying a GFP-expressing plasmid (PAO1-p*GFP*) (Figs. S6A and S6B). Fluorescence microscopy of log-phase PAO1Δ*fimL*+p*fimL*-*GFP* grown in the presence (data not shown) or absence of arabinose (Fig. 6D) revealed that FimL-GFP localized to both poles of the cell. Images of stationary phase PAO1Δ*fimL*+p*fimL*-*GFP* induced with arabinose show an apparent decrease in the frequency of polarly localized puncta (Fig. 6E), suggesting that overexpression of *fimL* leads to FimL protein delocalization and subsequent interference with function. Since overexpression of GFP fusion proteins can lead to artifactual localization [36], we replaced the native *fimL* locus with *fimL-gfp*. Expression of *fimL-GFP* from a single copy gene at the native locus restored TM and cAMP production (Figs. S6A and S6B) and exhibited polar localization regardless of growth phase (for representative images see Figs. 6A and 6B).

To examine whether CyaB is also polarly localized, we generated the strain PAO1Δ*cyaB* + p*cyaB*-*GFP*. With arabinose induction, TM was restored to wild type levels (Fig. S6C), and microscopy shows bipolar localization of CyaB-GFP and a strong signal around the perimeter of the cell in the absence (data not shown) or the presence of arabinose (Fig. 6F) from log-phase grown cells. Together, these results suggest that both FimL and CyaB are polarly localized.

## Discussion

cAMP is increasingly appreciated as an important regulator of diverse pathways in many bacteria. In *P. aeruginosa,* it is involved in the coordinate regulation of many critical virulence factors. CyaB, and a cAMP-dependent transcriptional regulator, Vfr, regulate the expression of over 200 genes, including components of the T3SS, TM, the T2SS, flagellar motility, and quorum sensing [12]. Through genetic and physiologic analyses, we demonstrate that FimL, a protein with two histidine phosphotransfer (Hpt) like domains, is required for optimum CyaB-dependent biosynthesis of cAMP. Additionally, we find that both FimL and CyaB are polarly localized, suggesting that spatial regulation of cAMP production may be important to the virulence of *P. aeruginosa*. Overexpression of *fimL* inhibits cAMP production and may cause FimL to mislocalize. We note that Fulcher *et al*. found that a disruption in *fimL* decreased cAMP levels in the *P. aeruginosa strain* PAK [29], suggesting this function in relevant across *Pseudomonad* species.

How might FimL contribute to cAMP production? Our experiments eliminate the possibility that FimL is required to maintain protein levels of CyaB in PAO1 or PA103, as no decrease in *cyaB* transcription or CyaB protein levels were observed in *fimL* mutants. Another possibility is that FimL enhances the adenylate cyclase activity of CyaB. In support of this hypothesis, epistasis experiments suggest that FimL and CyaB function in the same pathway. Our finding that both FimL and CyaB are polarly localized indicates that they may function as a complex. A simple hypothesis is that FimL is required for CyaB localization and/or CyaB is required for FimL localization. However, preliminary experiments suggest that their interactions may be more complicated, as FimL is still polarly localized in Δ*cyaB* mutants, and CyaB is polarly localized in the Δ*fimL* mutant (unpublished data). Interestingly, we have recently reported that CbpA, a cAMP binding protein of unknown function, is also localized to the poles [37]. An attractive possibility is that localized regulation of cAMP is important in regulating polar proteins. Indeed, spatial gradients of another second messenger signaling molecule, c-di-GMP, which is important for biofilm formation and swarming motility, have been recently reported [38].

**Figure 7:**
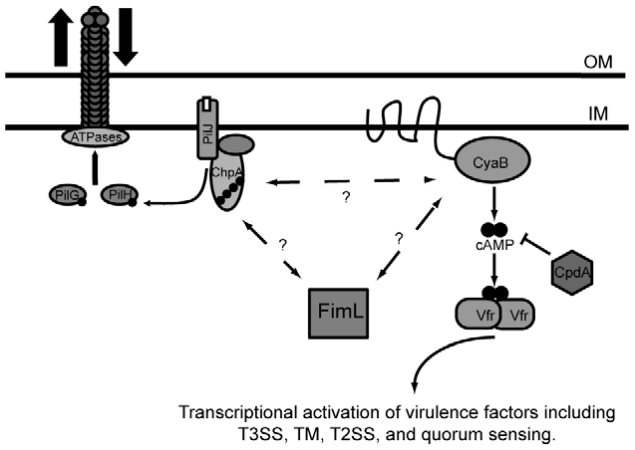
FimL may function to link the Chp system and CyaB to regulate cAMP levels in *P. aeruginosa*. FimL may interact with ChpA and/or CyaB to affect cAMP biosynthesis activity directly or indirectly. The Chp chemosensory system is thought to regulate pilus extension and retraction via two-component signaling. An input signal to the methyl-accepting chemotaxis protein PilJ induces autophosphorylation of the hybrid histidine kinase ChpA. PilG and PilH are putative response regulators that accept phosphoryl groups from phosphorylated ChpA and regulate pilus function. CpdA is a phosphodiesterase that degrades cAMP. cAMP is an allosteric regulator of Vfr which regulates multiple virulence pathways. OM and IM refer to the outer membrane and inner membrane respectively.

FimL shares homology with the N-terminal 563 amino acids of the 2477 amino acid ChpA protein, and it is tempting to speculate that binding interactions between FimL and ChpA or ChpA binding partners are important for regulating CyaB and/or other components of the cAMP regulatory circuit. ChpA is one of the most complex CheA homologs yet described, with 8 potential Hpt domains and a CheY-like domain [26]. Work from several labs suggests that the histidine kinase domain, the CheY domain, and at least two of the Hpt domains, are required for TM in PAO1 [32], [39]. Notably, glutamine (rather than histidine) is found at the two putative FimL phosphoryl group acceptor sites; hence it is unlikely that FimL is capable of phosphotransfer in a canonical manner. One possibility is that FimL, ChpA, and CyaB form a polarly localized complex to allow spatial, environmental, and temporal production of cAMP that regulates multiple polarly localized structures, including TFP and the T3SS (see Fig. 7).

ChpA is thought to be the central histidine kinase of a larger regulatory circuit with homology to chemosensory signal transduction systems. This module includes PilJ, a putative methyl-accepting chemotaxis protein; PilK, a methyltransferase homolog, 2 CheW homologs (PilI and ChpC); ChpB, a methylesterase homolog; and two additional CheY-like response regulators (PilG and PilH). PilJ, PilK, PilI, PilG, and PilH are required for TM. Fulcher and co-workers recently reported that disruption of *chpA*, *pilJ*, or *pilG* decreased cellular cAMP levels through a CyaB-dependent mechanism, whereas loss of *pilH* resulted in increased cAMP levels [29]. In conjunction with our finding that FimL also promotes cAMP production in a CyaB-dependent manner (this work) and that overexpression of either *fimL* (this work and [26]) or *chpA* [26] impairs cAMP-dependent pathways such as TM, we propose that FimL could link the Chp system and CyaB activation to promote cAMP synthesis (Fig. 7). This interaction may involve direct interactions between FimL and ChpA. Because ChpA contains 8 potential Hpt domains, there are many potential activation states that ChpA might occupy, allowing fine-tuning of response to environmental stimuli and subsequent downstream signaling. Interactions between FimL and ChpA could influence ChpA activity and affect downstream ChpA signaling.

An important strength of our studies is that we verified critical observations in two different strains. Our work demonstrates that in PAO1 and PA103, the cAMP defects in the Δ*fimL* and Δ*cyaB* mutants translate to defects in the Vfr-cAMP mediated functions, including decreased T3SS transcription and TFP-mediated motility. This finding contrasts the reported near wild type TM of PAKΔ*cyaB* [12] and PAKΔ*fimL* [25] despite the decreased cAMP levels in these mutants [29]. These differences suggest that additional or different factors may regulate TM in the PAK strain background. In addition, whereas *cyaB* transcription is reported in PAK to be induced under low calcium conditions [12], we observed that transcription and protein levels of CyaB were similar under calcium-poor and calcium-replete conditions.

We note some subtle differences between PAO1 and PA103 with respect to cAMP-mediated functions. *P_exoT_-lacZ* transcription was relatively insensitive to calcium in PA103, even though production and secretion of the T3SS effectors is induced in calcium-poor media. In contrast, *P_exoT_-lacZ* transcription in PAO1 was induced in media lacking calcium. These differences may reflect strain-specific fine-tuning of virulence circuits.

In summary, using a genetic screen and physiologic studies, we provide evidence that FimL regulates CyaB activity and cAMP production at a post-translational step and that both proteins exhibit polar localization. Future studies will be directed at determining the mechanism and role of the polarly localized cAMP biosynthesis and spatial regulation of virulence circuits.

## Supporting Information

Figure S1 Deletion of *cpdA* enhances *exoT* expression and TM in Δ*fimL*. (A) All strains harbor the P*exoT-lacZ* transcriptional reporter fusion as a read-out for transcription of the T3SS. β-galactosidase activity was measured in the presence or absence of Ca^2+^. Gene names denote in-frame deletions in PAO1. The results are normalized to wildtype values measured in the absence of Ca^2+^. Shown is the mean +/− SD of 12 data points of triplicate samples from 2 or 3 experiments. (B) Shown is the average colony diameter +/− SD from 8 colonies of a TM assay. (***) P<0.001, (**) P<0.01.
(TIF)

Figure S2 *fimL-3X-FLAG* restores cAMP production compared to Δ*fimL*.
(TIF)

Figure S3 Ectopic expression of p*fimL-FL* does not affect *cyaB* transcription. β-galactosidase activity was measured in the presence or absence of Ca^2+^ in PAO1 or PAO1 expressing p*fimL-FL*. Shown is the mean of three experiments. Error bars indicate SD.
(TIF)

Figure S4 Loss or ectopic expression of p*fimL* inhibits TM. Shown are representative colonies of the indicated strains. p denotes the empty vector and p*fimL*-FL denotes the vector with the *fimL*-FL insert.
(TIF)

Figure S5 Ectopic expression of *cyaB* restores P*exoT-lacZ* transcription in a Δf*imL* mutant. All strains harbor the P*exoT-lacZ* transcriptional reporter fusion integrated at the CTX site as a readout for transcription of the T3SS. β-galactosidase activity was measured in the presence or absence of Ca^2+^ in PAO1, PAO1Δ*cyaB*, or PAO1Δ*fimL* carrying the empty vector (p) or a vector with *cyaB* (p*cyaB*). Shown is the mean of 3 experiments performed in triplicate. Error bars indicate SD.
(TIF)

Figure S6 FimL-GFP is functional. (A) Colony diameter measurements showing TM in PAO1 and PAO1Δ*fimL*, with GFP plasmid (p*GFP*) or with pf*imL-GFP* grown in the presence or absence of the inducer arabinose. Arabinose induction of p*fimL-GFP* inhibits TM in PAO1 and in PAO1Δ*fimL*. Shown are the mean of at least 8 measurements. (B) cAMP measurements of the strains described in A. (C) Colony diameter measurements in Δ*cyaB* or Δ*cyaB* +p*cyaB-GFP*. Error bars indicate SD. (***) P<0.001 (*) P<0.5
(TIF)

Table S1 Strains and plasmids.
(DOC)
