# Supplementary figures and images for: FimL Regulates cAMP Synthesis in Pseudomonas aeruginosa
Source: PLoS One. 2011 Jan 11;6(1):e15867. doi: 10.1371/journal.pone.0015867 (PMC3019171; doi:10.1371/journal.pone.0015867)

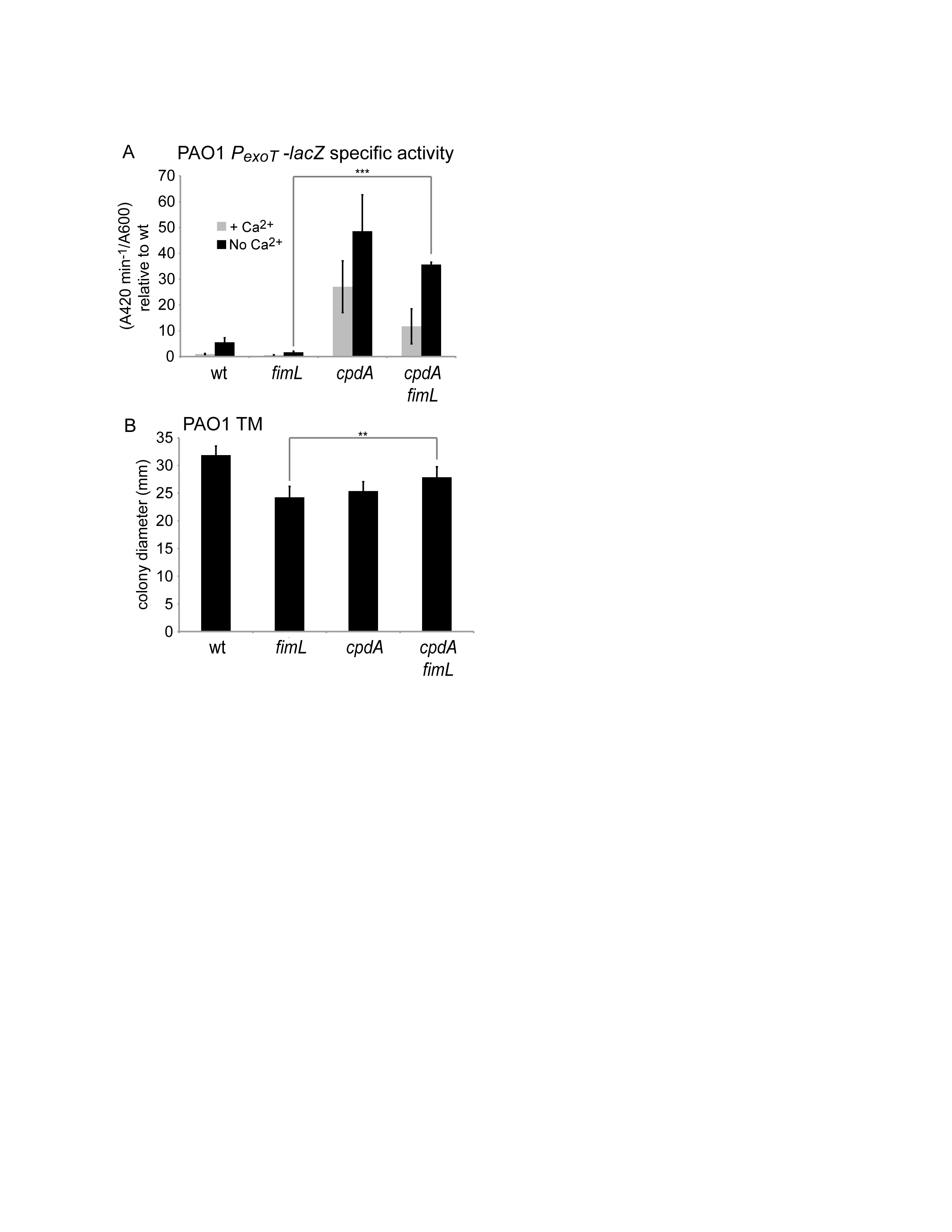

Supplement: Figure S1 — Deletion of cpdA enhances exoT expression and TM in ΔfimL. (A) All strains harbor the PexoT-lacZ transcriptional reporter fusion as a read-out for transcription of the T3SS. β-galactosidase activity was measured in the presence or absence of Ca2+. Gene names denote in-frame deletions in PAO1. The results are normalized to wildtype values measured in the absence of Ca2+. Shown is the mean +/− SD of 12 data points of triplicate samples from 2 or 3 experiments. (B) Shown is the average colony diameter +/− SD from 8 colonies of a TM assay. (***) P<0.001, (**) P<0.01. (TIF) [file pone.0015867.s001.tif]

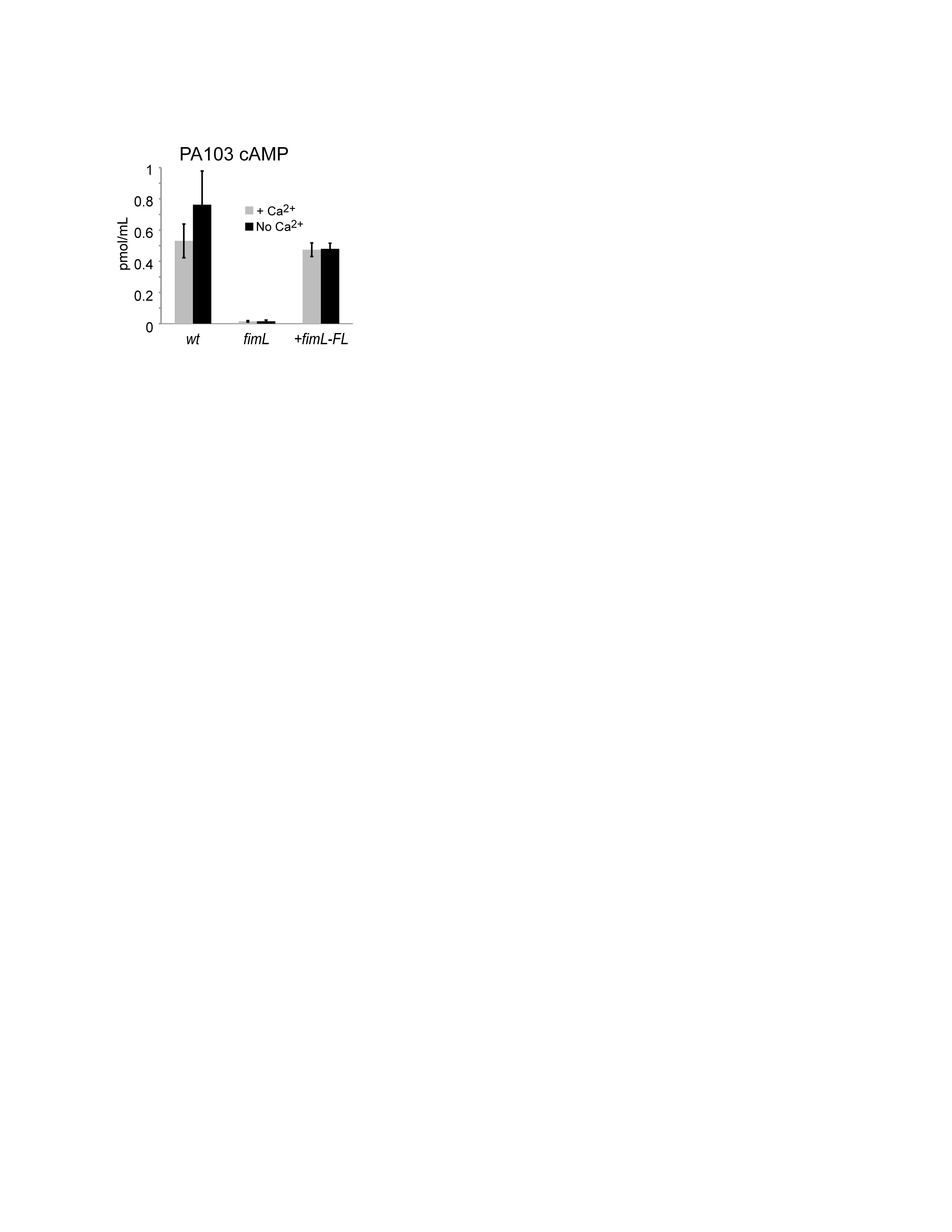

Supplement: Figure S2 — fimL-3X-FLAG restores cAMP production compared to ΔfimL. (TIF) [file pone.0015867.s002.tif]

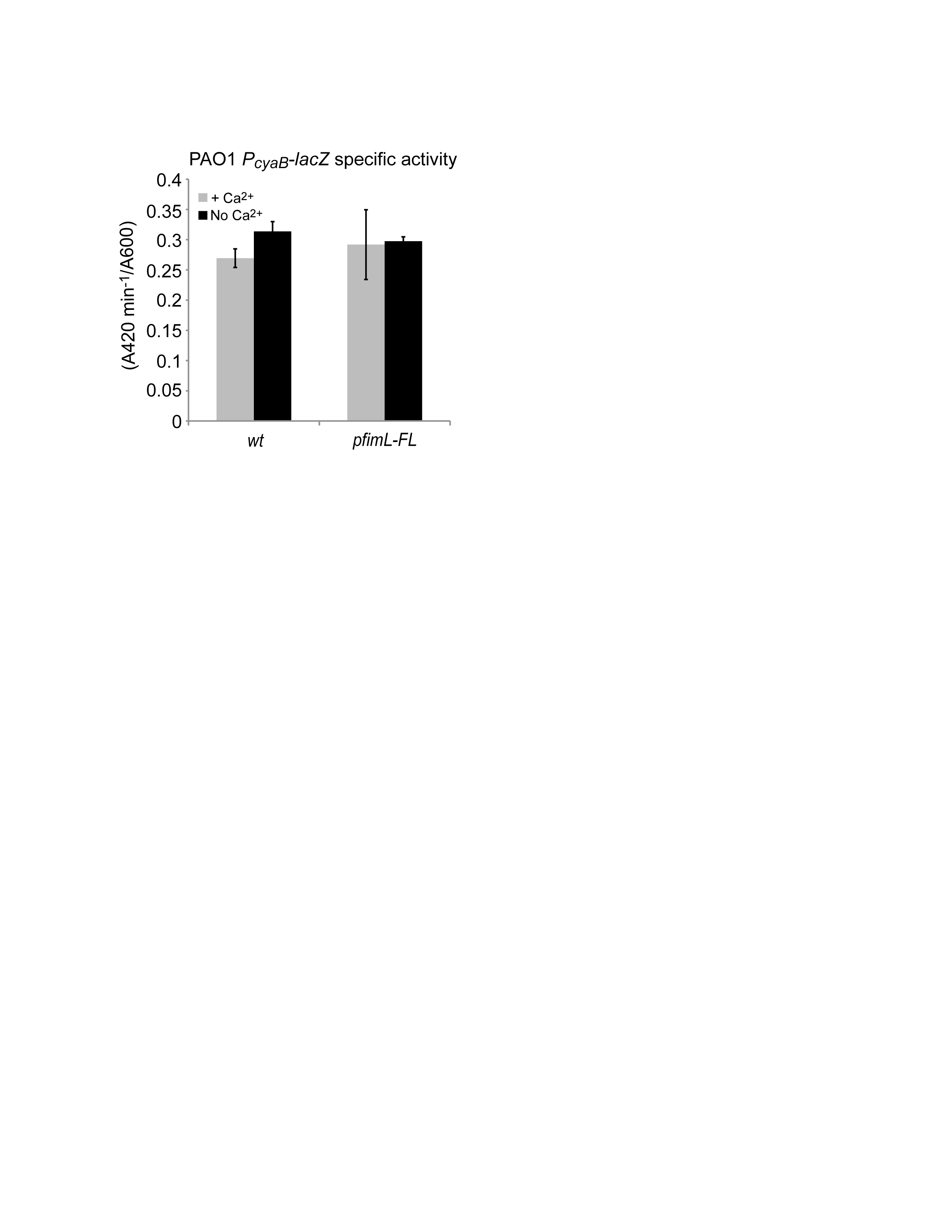

Supplement: Figure S3 — Ectopic expression of pfimL-FL does not affect cyaB transcription. β-galactosidase activity was measured in the presence or absence of Ca2+ in PAO1 or PAO1 expressing pfimL-FL. Shown is the mean of three experiments. Error bars indicate SD. (TIF) [file pone.0015867.s003.tif]

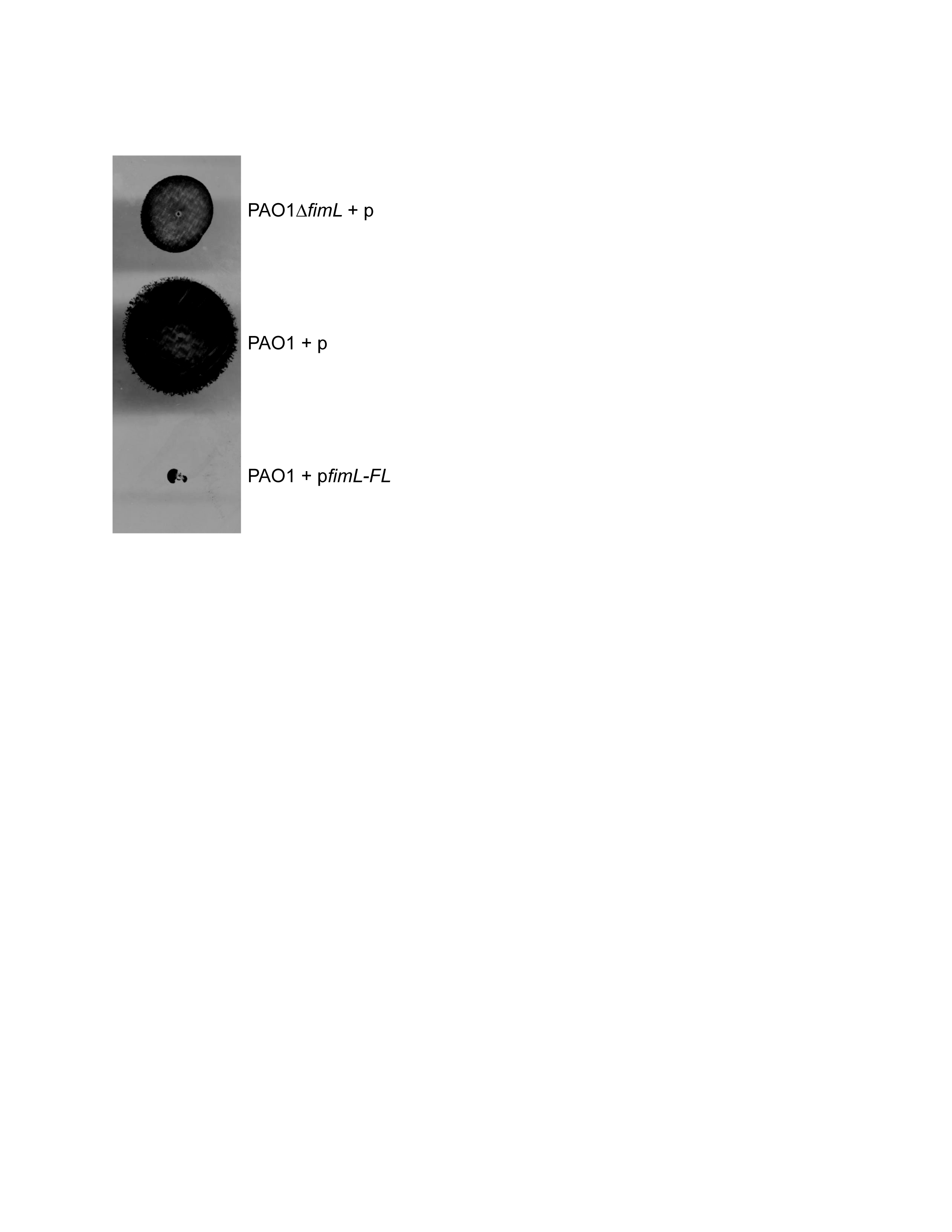

Supplement: Figure S4 — Loss or ectopic expression of pfimL inhibits TM. Shown are representative colonies of the indicated strains. p denotes the empty vector and pfimL-FL denotes the vector with the fimL-FL insert. (TIF) [file pone.0015867.s004.tif]

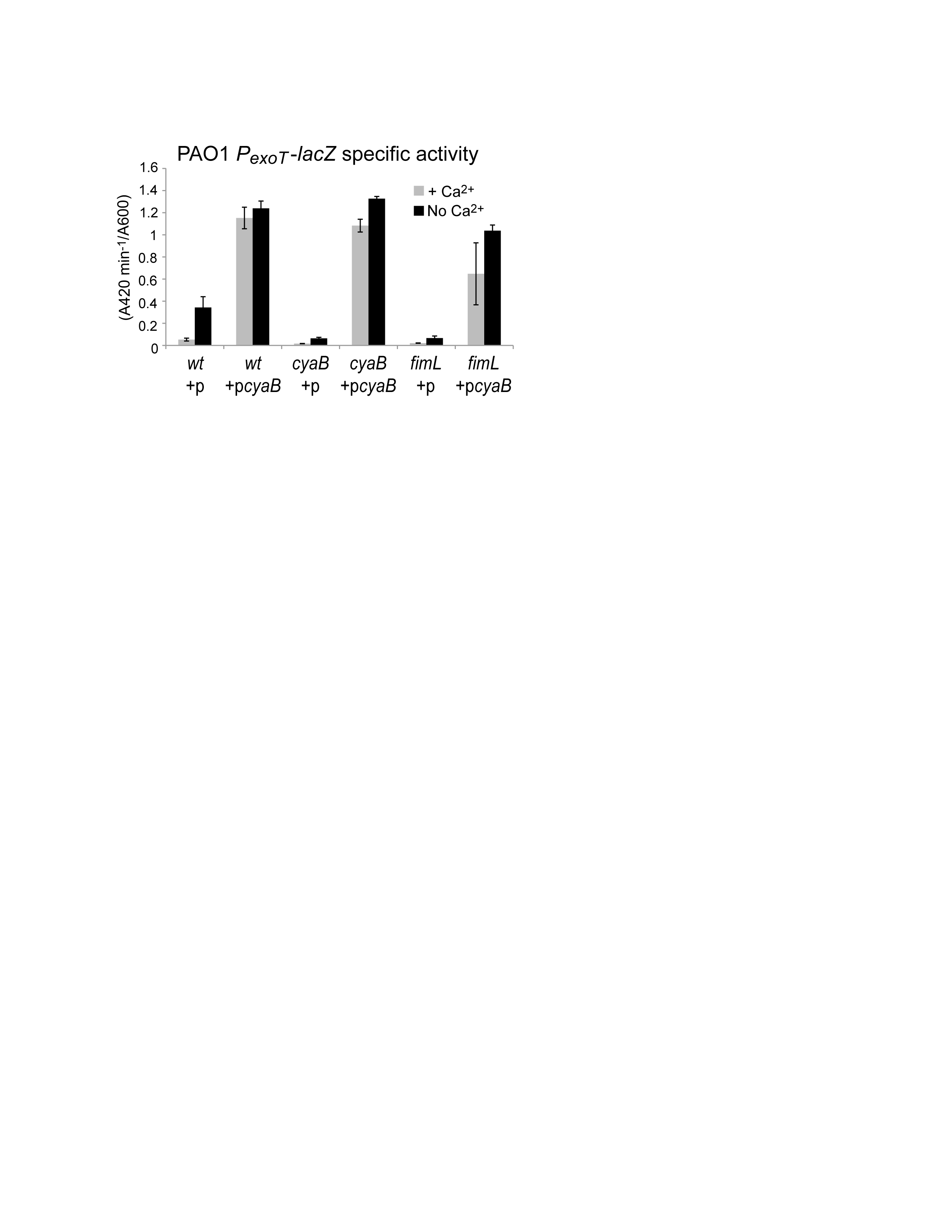

Supplement: Figure S5 — Ectopic expression of cyaB restores PexoT-lacZ transcription in a ΔfimL mutant. All strains harbor the PexoT-lacZ transcriptional reporter fusion integrated at the CTX site as a readout for transcription of the T3SS. β-galactosidase activity was measured in the presence or absence of Ca2+ in PAO1, PAO1ΔcyaB, or PAO1ΔfimL carrying the empty vector (p) or a vector with cyaB (pcyaB). Shown is the mean of 3 experiments performed in triplicate. Error bars indicate SD. (TIF) [file pone.0015867.s005.tif]

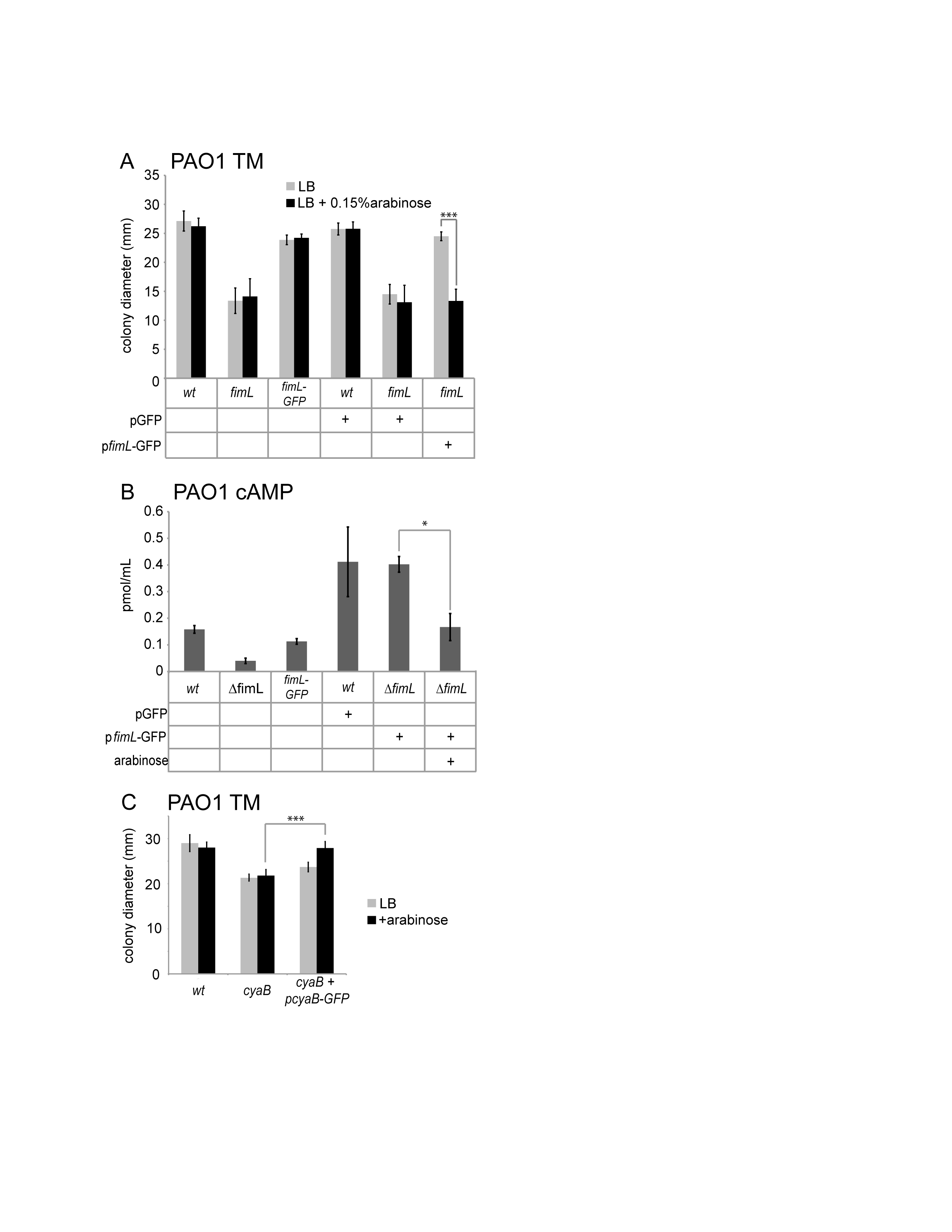

Supplement: Figure S6 — FimL-GFP is functional. (A) Colony diameter measurements showing TM in PAO1 and PAO1ΔfimL, with GFP plasmid (pGFP) or with pfimL-GFP grown in the presence or absence of the inducer arabinose. Arabinose induction of pfimL-GFP inhibits TM in PAO1 and in PAO1ΔfimL. Shown are the mean of at least 8 measurements. (B) cAMP measurements of the strains described in A. (C) Colony diameter measurements in ΔcyaB or ΔcyaB +pcyaB-GFP. Error bars indicate SD. (***) P<0.001 (*) P<0.5 (TIF) [file pone.0015867.s006.tif]
