# Supplementary material for: FimL Regulates cAMP Synthesis in Pseudomonas aeruginosa
Source: PLoS One. 2011 Jan 11;6(1):e15867. doi: 10.1371/journal.pone.0015867 (PMC3019171; doi:10.1371/journal.pone.0015867)
Supplement: Table S1 — Strains and plasmids. (DOC) [file pone.0015867.s007.doc]

| Table S1. Strains and plasmids | | |
| --- | --- | --- |
| Strain or plasmid | Genotype or relevant characteristics | Source or reference |
| *P. aeruginosa* |  |  |
| PAO1 | Wild type | [40] |
| PAO1*∆fimL* | In-frame deletion of *fimL* | [25] |
| PAO1*∆fimL*::CTX*-P_exoT_-lacZ* | In-frame deletion of *fimL* and *exoT* promoter fused to *lacZ* at the *attB* site | This study |
| PAO1*∆cpdA*::CTX-*P_exoT_-lacZ* | In-frame deletion of *cpdA* and *exoT* promoter fused to *lacZ* at the *attB* site | This study |
| PAO1*∆cpdA∆fimL*::CTX-*P_exoT_-lacZ* | In-frame deletion of *cpdA, fimL* and *exoT* promoter fused to *lacZ* at the *attB* site | This study |
| PAO1*∆cpdA∆cyaA*::CTX-*P_exoT_-lacZ* | In-frame deletion of *cpdA*, *cyaA* and *exoT* promoter fused to *lacZ* at the *attB* site | This study |
| PAO1*∆cpdA∆cyaB*::CTX-*P_exoT_-lacZ* | In-frame deletion of *cpdA*, *cyaB* and *exoT* promoter fused to *lacZ* at the *attB* site | This study |
| PAO1*∆cpdA∆cyaA∆cyaB*::CTX-*P_exoT_-lacZ* | In-frame deletion of *cpdA*, *cyaA*, *cyaB* and *exoT* promoter fused to *lacZ* at the *attB* site | This study |
| PAO1*∆cpdA∆cyaA∆fimL*::CTX-*P_exoT_-lacZ* | In-frame deletion of *cpdA*, *cyaA*, *fimL* and *exoT* promoter fused to *lacZ* at the *attB* site | This study |
| PAO1*∆cpdA∆cyaB∆fimL*::CTX-*P_exoT_-lacZ* | In-frame deletion of *cpdA*, *cyaB*, *fimL* and *exoT* promoter fused to *lacZ* at the *attB* site | This study |
| PAO1*∆cpdA fimL-FLAG*::CTX-*P_exoT_-lacZ* | In-frame deletion of *cpdA*, *fimL-3X-FLAG* and *exoT* promoter fused to *lacZ* at the *attB* site | This study |
| PAO1*∆cpdA∆cyaB*::CTX-*cyaB* | In-frame deletion of *cpdA*, *cyaB* and *cyaB* at the *attB* site (comp) | This study |
| PA103 | Wild-type | [41] |
| PA103::CTX-*P_exoT_-lacZ* | *exoT* promoter fused to *lacZ* at the *attB* site | This study |
| PA103*∆fimL*::CTX- *P_exoT_-lacZ* | In-frame deletion of *fimL* and *exoT* promoter fused to *lacZ* at the *attB* site | This study |
| PA103*∆cyaA*::CTX-*P_exoT_-lacZ* | In-frame deletion of *cyaA* and *exoT* promoter fused to *lacZ* at the *attB* site | This study |
| PA103*∆cyaB*::CTX-*P_exoT_-lacZ* | In-frame deletion of *cyaB* and *exoT* promoter fused to *lacZ* at the *attB* site | This study |
| PA103*∆cyaA∆cyaB*::CTX-*P_exoT_-lacZ* | In-frame deletion of *cyaA*, *cyaB* and *exoT* promoter fused to *lacZ* at the *attB* site | This study |
| PA103*∆vfr*::CTX-*P_exoT_-lacZ* | In-frame deletion of *vfr* and *exoT* promoter fused to *lacZ* at the *attB* site | This study |
| PA103 *fimL-FLAG exoT-HA*:: CTX-*P_exoT_-lacZ* | *fimL-3X-FLAG, exoT-HA* and *exoT* promoter fused to *lacZ* at the *attB* site | This study |
| PAO1::CTX*-lacZ* | *lacZ* at the *attB* site | CBW |
| PAO1::CTX*-P_cyaB_-lacZ* | *cyaB* promoter fused to *lacZ* at the *attB* site | CBW |
| PAO1*∆fimL*::CTX*-P_cyaB_-lacZ* | In-frame deletion of *fimL* and *cyaB* promoter fused to *lacZ* at the *attB* site | CBW |
| PAO1 *cyaB-HIS* | *cyaB-HIS* gene replacement | This study |
| PAO1*∆fimL* *cyaB-HIS* | In-frame deletion of *fimL* and *cyaB-HIS* gene replacement | This study |
| PAO1*∆vfr*::CTX- *P_exoT_-lacZ* | In-frame deletion of *vfr* and *exoT* promoter fused to *lacZ* at the *attB* site | This study |
| PAO1::CTX-*P_exoT_-lacZ* | *exoT* promoter fused to *lacZ* at the *attB* site | This study |
| PAO1*∆cyaA*::CTX-*P_exoT_-lacZ* | In-frame deletion of *cyaA* and *exoT* promoter fused to *lacZ* at the *attB* site | This study |
| PAO1*∆cyaB*::CTX-*P_exoT_-lacZ* | In-frame deletion of *cyaB* and *exoT* promoter fused to *lacZ* at the *attB* site | This study |
| PAO1*∆cyaA∆cyaB*::CTX-*P_exoT_-lacZ* | In-frame deletion of *cyaA*, *cyaB* and *exoT* promoter fused to *lacZ* at the *attB* site | This study |
| PAO1*∆cyaA∆fimL*::CTX-*P_exoT_-lacZ* | In-frame deletion of *cyaA*, *fimL* and *exoT* promoter fused to *lacZ* at the *attB* site | This study |
| PAO1*∆cyaB∆fimL*::CTX-*P_exoT_-lacZ* | In-frame deletion of *cyaB*, *fimL* and *exoT* promoter fused to *lacZ* at the *attB* site | This study |
| PAO1 *fimL-FLAG*::CTX-*P_exoT_-lacZ* | *fimL-3X-FLAG* gene replacement and *exoT* promoter fused to *lacZ* at the *attB* site | This study |
| PAO1*∆cyaB*::CTX-*cyaB* | In-frame deletion of *cyaB* and *cyaB* at the *attB* site (comp) | This study |
| PA103*∆cyaA∆fimL*::CTX-*P_exoT_-lacZ* | In-frame deletion of *cyaA*, *fimL* and *exoT* promoter fused to *lacZ* at the *attB* site | This study |
| PA103*∆cyaB∆fimL*::CTX-*P_exoT_-lacZ* | In-frame deletion of *cyaB*, *fimL* and *exoT* promoter fused to *lacZ* at the *attB* site | This study |
| PA103*∆cyaB*::CTX-*cyaB* | In-frame deletion of *cyaB* and *cyaB* at the *attB* site (comp) | This study |
| PAO1*∆cpdA∆vfr*::CTX-*P_exoT_-lacZ* | In-frame deletion of *cpdA*, *vfr* and *exoT* promoter fused to *lacZ* at the *attB* site | This study |
| PAO1*∆cpdA*::CTX-*P_exoT_-lacZ* +pUCP19*∆lac* | In-frame deletion of *cpdA* and *exoT* promoter fused to *lacZ* at the *attB* site with pUCP19*∆lac* | This study |
| PAO1*∆cpdA*::CTX-*P_exoT_-lacZ* +pJTW017 | In-frame deletion of *cpdA* and *exoT* promoter fused to *lacZ* at the *attB* site with pUCP19*∆lac* carrying *fimL-3X-FLAG* | This study |
| PAO1*∆cpdA∆cyaA*::CTX-*P_exoT_-lacZ* +pUCP19*∆lac* | In-frame deletion of *cpdA, cyaA* and *exoT* promoter fused to *lacZ* at the *attB* site with pUCP19*∆lac* | This study |
| PAO1*∆cpdA∆cyaA*::CTX-*P_exoT_-lacZ* + pJTW017 | In-frame deletion of *cpdA, cyaA* and *exoT* promoter fused to *lacZ* at the *attB* site with pUCP19*∆lac* carrying *fimL-3X-FLAG* | This study |
| PAO1*∆cpdA∆cyaB*::CTX-*P_exoT_-lacZ* +pUCP19*∆lac* | In-frame deletion of *cpdA, cyaB* and *exoT* promoter fused to *lacZ* at the *attB* site with pUCP19*∆lac* | This study |
| PAO1*∆cpdA∆cyaB*::CTX-*P_exoT_-lacZ* + pJTW017 | In-frame deletion of *cpdA, cyaB* and *exoT* promoter fused to *lacZ* at the *attB* site with pUCP19*∆lac* carrying *fimL-3X-FLAG* | This study |
| PA103::CTX-*P_exoT_-lacZ* +pUCP19*∆lac* | *exoT* promoter fused to *lacZ* at the *attB* site with pUCP19*∆lac* | This study |
| PA103::CTX-*P_exoT_-lacZ* +pJTW017 | *exoT* promoter fused to *lacZ* at the *attB* site with pUCP19*∆lac* carrying *fimL-3X-FLAG* | This study |
| PA103*∆cyaA*::CTX-*P_exoT_-lacZ* +pUCP19*∆lac* | In-frame deletion of *cyaA* and *exoT* promoter fused to *lacZ* at the *attB* site with pUCP19*∆lac* | This study |
| PA103*∆cyaA*::CTX-*P_exoT_-lacZ* + pJTW017 | In-frame deletion of *cyaA* and *exoT* promoter fused to *lacZ* at the *attB* site with pUCP19*∆lac* carrying *fimL-3X-FLAG* | This study |
| PA103*∆cyaB*::CTX-*P_exoT_-lacZ* +pUCP19*∆lac* | In-frame deletion of *cyaB* and *exoT* promoter fused to *lacZ* at the *attB* site with pUCP19*∆lac* | This study |
| PA103*∆cyaB*::CTX-*P_exoT_-lacZ* + pJTW017 | In-frame deletion of *cyaB* and *exoT* promoter fused to *lacZ* at the *attB* site with pUCP19*∆lac* carrying *fimL-3X-FLAG* | This study |
| PAO1+pMBAD-GFP | Plasmid pMBAD-GFP, Gm^r^ | [37] |
| PAO1+pMBAD-FimL-GFP | pMBAD-GFP carrying *fimL-GFP,* Gm^r^ | This study |
| PAO1*∆fimL*+pMBAD-GFP | In-frame deletion of *fimL* with plasmid pMBAD-GFP, Gm^r^ | This study |
| PAO1*∆fimL*+pMBAD-FimL-GFP | In-frame deletion of fimL with pMBAD-GFP carrying *fimL-GFP,* Gm^r^ |  |
| PAO1 *fimL-GFP* | *fimL-GFP* gene replacement | This study |
| PAO1::CTX*-P_cyaB_-lacZ*+pJTW017 | *cyaB* promoter fused to *lacZ* at the *attB* site with pUCP19*∆lac* carrying *fimL-3X-FLAG,* Cb^r^ | This study |
| PAO1::CTX-*P_exoT_-lacZ* + pUCP19*∆lac* | *exoT* promoter fused to *lacZ* at the *attB* site with pUCP19*∆lac*, Cb^r^ | This study |
| PAO1*∆fimL*::CTX-*P_exoT_-lacZ* + pUCP19*∆lac* | In-frame deletion of *fimL* and *exoT* promoter fused to *lacZ* at the *attB* site with pUCP19*∆lac*, Cb^r^ | This study |
| PAO1::CTX-*P_exoT_-lacZ* + pJTW017 | *exoT* promoter fused to *lacZ* at the *attB* site with pUCP19*∆lac* carrying *fimL-3X-FLAG*, Cb^r^ | This study |
| PAO1::CTX-*P_exoT_-lacZ*+pJTW093 | *exoT* promoter fused to *lacZ* at the *attB* site with pUCP19*∆lac* carrying *cyaB*, Cb^r^ | This study |
| PAO1*∆cyaB*::CTX-*P_exoT_-lacZ*+ pUCP19*∆lac* | In-frame deletion of *cyaB* and *exoT* promoter fused to *lacZ* at the *attB* site with pUCP19*∆lac*, Cb^r^ | This study |
| PAO1*∆cyaB*::CTX-*P_exoT_-lacZ*+ pJTW093 | In-frame deletion of *cyaB* and *exoT* promoter fused to *lacZ* at the *attB* site with pUCP19*∆lac* carrying *cyaB*, Cb^r^ | This study |
| PAO1*∆fimL*::CTX-*P_exoT_-lacZ*+ pUCP19*∆lac* | In-frame deletion of *fimL* and *exoT* promoter fused to *lacZ* at the *attB* site with pUCP19*∆lac*, Cb^r^ | This study |
| PAO1*∆fimL*::CTX-*P_exoT_-lacZ*+ pJTW093 | In-frame deletion of *fimL* and *exoT* promoter fused to *lacZ* at the *attB* site with pUCP19*∆lac* carrying *cyaB*, Cb^r^ | This study |
|  |  |  |
| *E. coli* |  |  |
| DH5α | *hsdR* *rec* l*acZYA* φ80 *lacZ*M15 | Invitrogen |
| S17.1λ*pir* | *thi pro hsdR recA* *RP4-2*(Tc::Mu)(Km::Tn7) | Stratagene |
| SM10λ*pir* | *thi thr leu tonA lacY supE recA::RP4-2-Tc::Mu Km* | [42] |
|  |  |  |
| Plasmids |  |  |
| pBT20 | Contains mariner transposon, Gm^r^ | [33] |
| mini-CTX-*lacZ* | Contains promoterless *lacZ* for integration at the *attB* site on the *P. aeruginosa* chromosome, Tc^r^ | [43] |
| mini-CTX2 | Construct for introduction of exogenous DNA fragments at the *attB* site, Tc^r^ | [43] |
| pFLP2 | Source of Flp recombinase; Ap^r^ | [44] |
| pOK12 | *E. coli* cloning vector; Km^r^ | [45] |
| pEX100T | Allelic replacement suicide plasmid; Ap^r^ (Cb^r^) | [46] |
| pJEN36 | pEX100T with *∆fimL* deletion construct, Ap^r^ (Cb^r^) | [26] |
| pJEN51 | pEX100T with *∆vfr* deletion construct, Ap^r^ (Cb^r^) | [26] |
| pMBAD-GFP | C-terminal GFP fusion construct, Gm^r^ | [37] |
| pYFI007 | pMBAD-GFP containing *fimL*, Gm^r^ | This study |
| pJB100T | pEX100T derivative replacing SmaI site with the SpeI site, Ap^r^ (Cb^r^) | [32] |
| pYFI043 | pJB100T carrying *fimL-GFP* gene replacement construct, Ap^r^ (Cb^r^) | This study |
| pJTW031 | pEX100T carrying *∆cyaB* deletion construct, Ap^r^ (Cb^r^) | This study |
| pUCP19*∆lac* | pUCP19 derivative in which *lacI* has been deleted, Ap^r^ (Cb^r^) | JJB |
| pJTW093 | pUCP19*∆lac* carrying *cyaB*, Ap^r^ (Cb^r^) | This study |
| pJTW017 | pUCP19*∆lac* carrying *fimL-3X-FLAG*, Ap^r^ (Cb^r^) | This study |
| pJTW019 | pEX100T carrying *fimL-3X-FLAG* replacement construct, Ap^r^ (Cb^r^) | This study |
| pJTW091 | Mini-CTX-*lacZ* carrying *exoT* promoter to construct P*_exoT_-lacZ*, Tc^r^ | This study |
| pJTW198 | Mini-CTX-*lacZ* carrying *cyaB* promoter to construct P*_cyaB_-lacZ*, Tc^r^ | This study |
| pJTW033 | pEX100T carrying *∆cpdA* deletion construct, Ap^r^ (Cb^r^) | This study |
| pJTW032 | pEX100T carrying *∆cyaA* deletion construct, Ap^r^ (Cb^r^) | This study |
| pJTW196 | Mini-CTX2 with *cyaB*, Tc^r^ | This study |
| pJTW053 | pEX100T carrying *cyaB-His* gene replacement construct, Ap^r^ (Cb^r^) | This study |
| pYFI184 | pMBAD-GFP containing *cyaB*, Gm^r^ | This study |
|  |  |  |

Gm^r^, gentamicin; Tc^r^, tetracycline; Cb^r^, carbenicillin; Km^r^, kanamycin; Ap^r^, ampicillin
